# Supplementary material for: Solar cell designs by maximizing energy production based on machine learning clustering of spectral variations
Source: Nat Commun. 2018 Dec 3;9:5126. doi: 10.1038/s41467-018-07431-3 (PMC6277435; doi:10.1038/s41467-018-07431-3)
Supplement: Supplementary file 1 — Supplementary Information [file 41467_2018_7431_MOESM1_ESM.pdf]

Supplementary Information for "Solar cell designs maximizing annual energy production based on machine learning of spectral variations"

J. M. Ripalda[1,*], J. Buencuerpo[1,2], and I. García[3]

[1]Instituto de Micro y Nanotecnología, IMN-CNM, CSIC (CEI UAM+CSIC) Isaac Newton, 8, E-28760, Tres Cantos, Madrid, Spain
[*]j.ripalda@csic.es
[2]Now at National Renewable Energy Laboratory, Golden, Colorado 80401, USA
[3]Instituto de Energía Solar, Universidad Politécnica de Madrid, Avda. Complutense 30, 28040, Madrid, Spain

Supplementary Figures

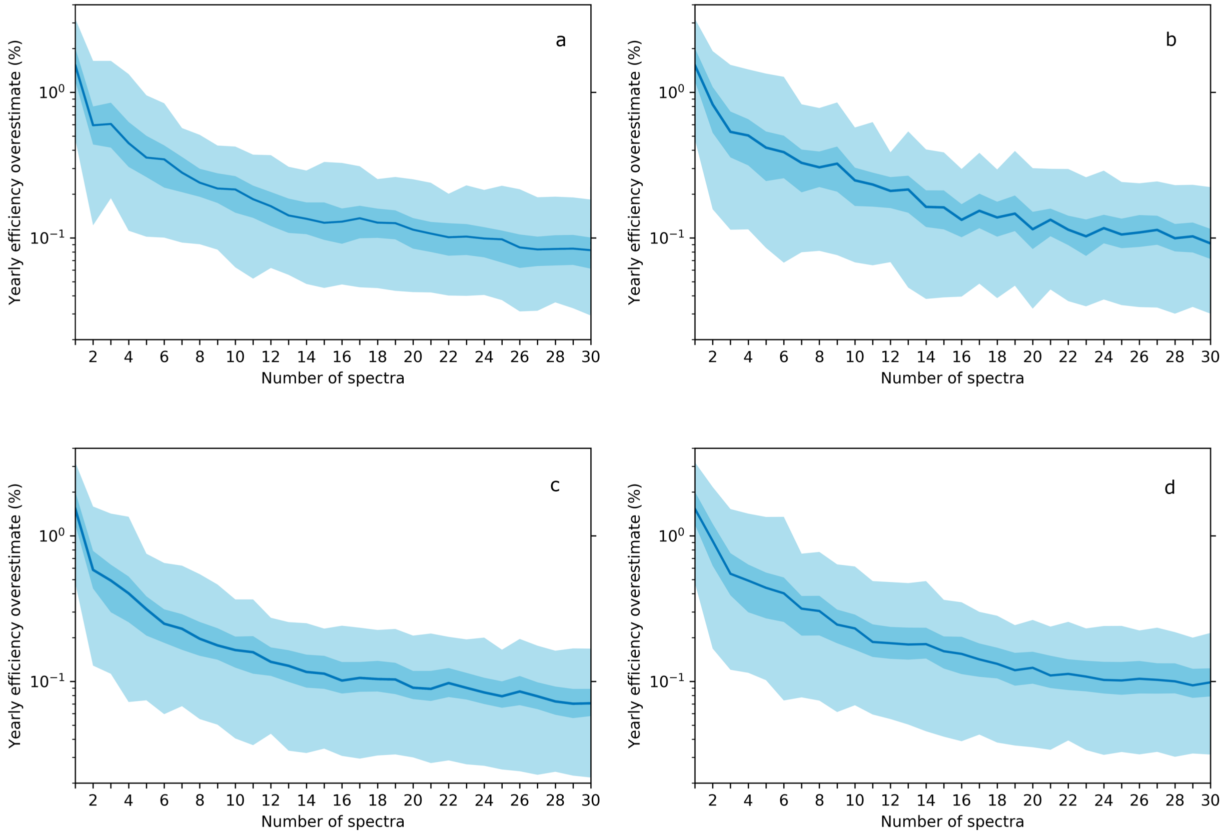

Supplementary Figure 1 *k*-means and related methods. Convergence of the yearly averaged efficiency as a function of the number of proxy spectra. The data corresponds to a set of random, but near optimal (within 2% of the maximum efficiency), series connected 6 junction devices. The middle line is the median, and the shadowed areas correspond to the quartile ranges, *i.e.*: min. to first quartile (Q1), Q1 to median, median to third quartile (Q3), and Q3 to max. **(a)** Spectral Clustering. **(b)** Mini batch *k*-means. **(c)** *k*-means with post-filtering of small clusters. **(d)** *k*-means.

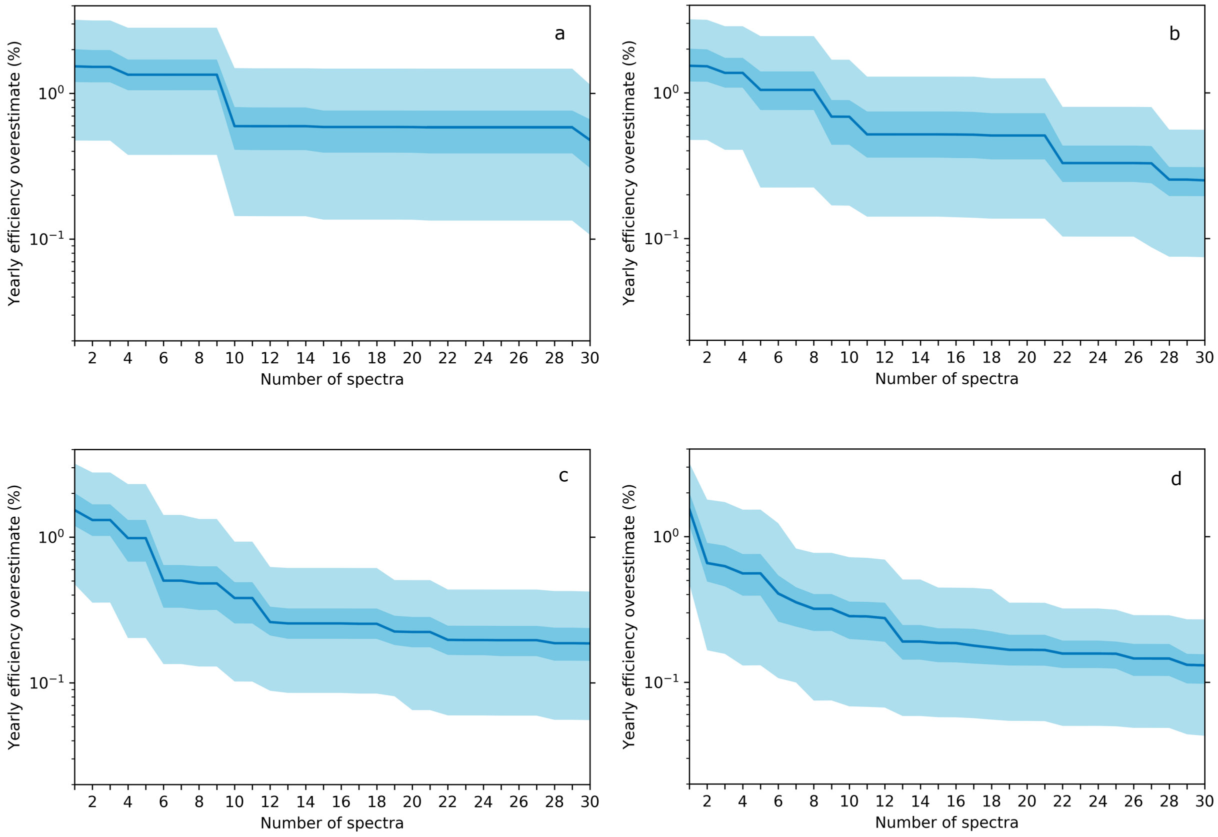

Supplementary Figure 2 Agglomerative clustering and related methods. Convergence of the yearly averaged efficiency as a function of the number of proxy spectra. The data corresponds to a set of random, but near optimal (within 2% of the maximum efficiency), series connected 6 junction devices. The middle line is the median, and the shadowed areas correspond to the quartile ranges, *i.e.*: min. to first quartile (Q1), Q1 to median, median to third quartile (Q3), and Q3 to max. **(a)** Agglomerative clustering with average linkage. **(b)** Agglomerative clustering with complete linkage. **(c)** Agglomerative clustering with Ward linkage. **(d)** Birch.

Supplementary Tables

Supplementary Table 1 Comparison of the median and the maximum efficiency overestimate, and relative computational cost for various clustering methods.

Method	Median (%)	Max. (%)	Relative comp. cost
k-means with post-filtering	0.07	0.17	79.0
k-means	0.08	0.22	57.3
Spectral clustering	0.08	0.18	1168.1
Mini-batch *k*-means	0.09	0.22	4.7
Aggl. Ward linkage	0.13	0.27	154.2
Binning	0.13	0.71	1.0
Birch	0.19	0.42	12.0
Aggl. complete linkage	0.25	0.56	153.0
Aggl. average linkage	0.48	1.15	154.4

Supplementary Notes

Supplementary Note 1: Other machine learning methods

We have focused our efforts on unsupervised machine learning techniques. Supervised machine learning methods such as support vector machines and neural networks might be useful for this application, but are not directly comparable to the unsupervised methods here used as they would have required major changes to how we initially designed our study. There are many unsupervised machine learning methods described in the literature, many more than we would have had time to consider, so we have constrained our study to the methods found in the sklearn open source machine learning library. Some of these methods (such as mean shift and affinity propagation) are known to be non-scalable with the number of samples, i.e.: not appropriate for a large number of input spectra. Other methods, such as DBSCAN, are known to be most suitable for applications where clusters are separated by areas of low density of samples, which is not the case here. Furthermore, these three methods (DBSCAN, affinity propagation, and mean shift) do not allow for direct control of the number of clusters, instead the number of resulting clusters depends indirectly on the fine tuning of the free parameters of the method. As a consequence of these difficulties, these three methods have been excluded from our study after some preliminary testing.

Supplementary Note 2: Comparison of clustering methods

For most of the tested methods, with the exception of *k*-means and spectral clustering (Supplementary Figure 1), the resulting cluster sizes are extremely in-homogeneous with a large fraction of the spectra accumulated in a few clusters, regardless of the total number of clusters. As a consequence, the error margin of these methods decreases slowly as the number of clusters is increased. This is most obvious in the case of agglomerative clustering (Supplementary Figure 2).

In Supplementary Table 1 we compare the resulting median and maximum efficiency overestimate for each clustering method. The quoted results are for sets of 30 proxy spectra. The yearly averaged efficiencies for a set of random but nearly optimal (within 2% of the maximum efficiency) 6-junction series connected solar cells are calculated using the full spectral set of 2×10^4 spectra, and compared with the results obtained with sets of proxy spectra obtained from various methods. The relative computational cost is normalized to the cost of the binning method. In all cases we have used the same previous steps of spectra normalization, feature agglomeration, classification, and restoration of the spectra to their initial values, as described in the methodology section of the main manuscript.
